# *Notes from the Field*: Rabies Outbreak Investigation — Pedernales, Dominican Republic, 2019

**DOI:** 10.15585/mmwr.mm6832a5

**Published:** 2019-08-16

**Authors:** Anna Mandra, David Morán, Patricia Valerio Santana, Miguel de la Cruz Marrero, Elina Díaz, Manuel Gil, Rannily Rojas Nolasco, Rinaldy Capellan, Xiomara Acosta, Rafael Pérez, Carmen Céspedes, Bienvenido Báez, Rene Edgar Condori, Todd Smith, James Ellison, Lauren Greenberg, Benjamin Monroe, Andy Gibson, Ryan M. Wallace, Brett Petersen

**Affiliations:** ^1^Division of High-Consequence Pathogens and Pathology, National Center for Emerging and Zoonotic Infectious Diseases, CDC; ^2^Epidemic Intelligence Service, CDC; ^3^Division of Global Health Protection, Center for Global Health, Guatemala Country Office, CDC; ^4^Centro de Prevención y Control de Enfermedades Transmitidas por Vectores y Zoonosis, Ministerio de Salud Pública, Santo Domingo, Dominican Republic; ^5^Dirección Provincial de Salud Pedernales, Ministerio de Salud Pública, Santo Domingo, Dominican Republic; ^6^Field Epidemiology Training Program, Dirección General de Epidemiología, Ministerio de Salud Pública, Santo Domingo, Dominican Republic; ^7^Mission Rabies, Dorset, United Kingdom.

On July 13, 2018, a child from Pedernales, Dominican Republic, died after developing clinical signs and symptoms consistent with rabies. Because of the child’s signs and symptoms, history of having been bitten by a dog 4 months earlier, and not having a received postexposure prophylaxis (PEP) (*1*), the patient was reported as having a probable case of rabies to the Ministerio de Salud Pública (MSP; i.e., Ministry of Public Health) (*1*). This case was the first reported from Pedernales Province in >30 years. During November 29–December 20, 2018, two additional probable rabies cases (based on clinical signs and history of dog bites) in children were reported from this province. The second patient did not receive any PEP. The third patient began PEP 10 days after being bitten and received 4 doses of vaccine before symptom onset; no rabies immunoglobulin was available in the province. All three children died from rabies encephalitis.

All three cases were confirmed by detection of rabies-specific antigen and nucleic acid in patients’ biologic specimens by direct florescent antibody and real-time reverse transcription–polymerase chain reaction testing at CDC (*1*). Complete nucleoprotein gene sequencing revealed a canine rabies virus variant. Three reported human rabies cases in 6 months exceeded the national average of zero to one per year (*2*). Because Pedernales borders Anse-à-Pitre, Haiti, and mixing of canine populations occurs, a binational coordinated response was initiated (*3*). This report focuses on the Dominican Republic response. At MSP’s request, CDC assisted with an outbreak investigation focused on active surveillance for animal bites and canine and human rabies cases, evaluation of canine vaccination coverage, and verification of the potency of human and veterinary rabies vaccines. Because it was an emergency outbreak response, the investigation was determined to be nonresearch.

Hospital animal bite records, animal investigations, and medical records were reviewed, and 224 households were surveyed to 1) identify probable animal rabies cases and animal bites to humans and 2) estimate the number of always-confined and sometimes-confined dogs. During January 2018–January 2019, a total of 29 probable animal rabies cases and 387 animal bites to humans were reported to MSP (Figure). Included in the 387 reported animal bites were 31 of the 39 bites identified by the household survey; patients who received eight (21%) of the 39 bites did not seek medical care and would not have been found by routine surveillance. Untreated bites were assessed to ascertain risk for rabies and whether PEP would have been recommended; five of the patients involved in the eight previously unidentified bites did not require PEP because the animal was alive 10 days after the bite. No evidence of unreported deaths consistent with rabies was found.

**FIGURE:**
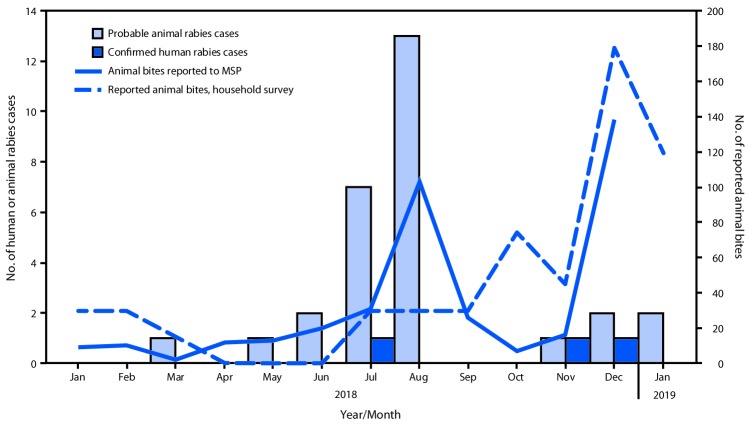
Reported animal bites,* confirmed human rabies cases, and probable animal rabies cases — Pedernales, Dominican Republic, January 2018–January 2019 **Abbreviation:** MSP = Ministerio de Salud (Ministry of Health). * Animal bites reported to MSP include 31 bites reported in household survey (eight of 39 bites identified in survey were not reported to MSP because patients bitten did not seek treatment).

Before this investigation began on January 28, 2019, a human-to-dog ratio of six to one was used to calculate dog population size and the number of vaccine doses required to reach 70% coverage; the total provincial dog population was estimated to be 5,678. During August 9–27, 2018, MSP conducted a door-to-door canine vaccination campaign and vaccinated 4,099 dogs (72% of the estimated total dog population) using a locally produced canine vaccine. However, a vaccine from a different manufacturer was used to vaccinate an additional 231 dogs during an emergency campaign that was initiated after the second case of human rabies and was ongoing at the time of the investigation.

Because cases of human rabies occurred after the door-to-door dog vaccination campaign in August 2018, population size categorized by roaming status (always-confined, sometimes-confined, and always–free-roaming) was calculated to evaluate vaccination coverage. The always–free-roaming dog population was estimated using a sight-resight methodology (similar to capture-recapture) and a mobile phone application created by Mission Rabies (http://www.missionrabies.com/). Numbers of always-confined and sometimes-confined dogs were obtained from the household survey.

The numbers of sometimes-confined and always–free-roaming dogs was higher than those in previous estimates, resulting in a human-to-dog ratio of 3.39 to one (95% confidence interval = 3.04–3.82; estimated total province dog population = 8,872–11,207). Because the campaign used door-to-door vaccination, which targets always-confined and sometimes-confined dogs, the population of always–free-roaming dogs likely was not adequately reached during the door-to-door vaccination campaign, resulting in ongoing transmission.

To assess canine rabies vaccine potency, available vaccines were collected; because batches used during the campaign were unavailable, serum from eight vaccinated dogs was collected to measure antibody titers as an indicator of vaccine potency. An antigen-capture electrochemiluminescent assay at CDC was used to evaluate available human and veterinary vaccines (*4*). Vaccines tested were similar to known potent reference vaccines and predicted to be potent. Eight serum samples from vaccinated dogs were tested for rabies virus–neutralizing antibody titer by rapid fluorescent focus inhibition test; two had a passing titer ≥0.5 IU/mL, and all displayed complete neutralization at 1:5 at 6 months postvaccination, demonstrating prior vaccination.

CDC recommended to MSP that before future campaigns, the estimated total dog population size and roaming status be evaluated. Vaccination strategies (e.g., door-to-door, capture-vaccinate-release) can be adapted to achieve 70% annual vaccination coverage in all canine population categories (*5*). Because of the close association between the border towns of Pedernales, Dominican Republic, and Anse-à-Pitre, Haiti, and the mixing of the canine populations, binational coordination for rabies control needs to continue (*3*).
